# The association of metabolic syndrome and its factors with gallstone disease

**DOI:** 10.1186/1471-2296-15-138

**Published:** 2014-07-29

**Authors:** I-Ching Lin, Yu-Wen Yang, Mei-Feng Wu, Yi-Hui Yeh, Jenn-Chang Liou, Ying-Li Lin, Chih-Hsiang Chiang

**Affiliations:** 1 Family Medicine Department, Changhua Christian Hospital, No. 135, St. Nan-Xiao, Changhua City 500, Taiwan; 2 Department of Healthcare Administration, Asia University, Taichung City, Taiwan; 3 School of Medicine, Chung Shan Medical University, Taichung City, Taiwan; 4 Health Examination Department, Changhua Christian Hospital, Changhua City, Taiwan; 5 Institute of Statistics, National Chung Hsing University, Taichung City, Taiwan

**Keywords:** Obesity, Dyslipidemia, Metabolic factors, Metabolic syndrome, Gallstone disease

## Abstract

**Background:**

To investigate the association between metabolic syndrome, including its factors, and gallstone disease (GSD) in a Taiwanese population.

**Methods:**

We conducted a cross-sectional study during 2011 ~ 2012. A total of 12050 subjects who completed a questionnaire and underwent physical examination, laboratory tests and abdominal ultrasonography formed the study population.

**Results:**

The prevalences of metabolic syndrome and gallstone disease were 24.09% and 6.16%. In an age- and sex-adjusted logistic regression model, metabolic syndrome was associated with gallstone disease (OR = 1.61; P < 0.0001). Age, abdominal obesity, and lower high-density lipoprotein cholesterol were associated with gallstone disease after adjusting for other factors. Females had a higher odds ratio than males in waist circumference for GSD, whereas males had a lower odds ratio than females in HDL-C for GSD.

**Conclusions:**

The present study suggests that metabolic syndrome is related to gallstone disease. Waist circumference and high-density lipoprotein cholesterol are all associated with GSD. Men and women may possibly have different priorities and strategies to reduce the burden of GSD.

## Background

Metabolic syndrome (MetS) means the presence of multiple risk factors for cardiovascular disease (CVD). The report from the National Cholesterol Education Program’s Adult Treatment Panel III (ATP III) identified 6 components of metabolic syndrome that are related to CVD: abdominal obesity, atherogenic dyslipidemia, raised blood pressure, insulin resistance, glucose intolerance, a pro-inflammatory state and a prothrombotic state [1]. MetS increases the risks of atherosclerosis and CVD. Some studies have also suggested that it may be related to some gastrointestinal tract diseases, such as fatty liver, nonalcoholic steatohepatitis, and unexplained liver cirrhosis [2].

Gallstone disease (GSD) is a chronic disease that consumes a lot of economic and medical resources [3]. It not only affects patients’ life quality, but also is associated with the potential risks of the development of cholecystitis, pancreatitis, biliary tract obstruction and gall bladder cancer [2,4]. In Taiwan, the overall prevalence of gallstone disease is around 4.3 - 10.7% [4,5].

If the risk factors of GSD can be identified, more interventions can be introduced earlier to prevent the disease. According to studies in Western countries, sex, age, body mass index (BMI), hyperlipidemia, usage of oral contraceptives, alcohol consumption, diabetes mellitus (DM), and race have been reported to be closely related to GSD [6,7]. Among these, hyperlipidemia, obesity and diabetes mellitus (DM) are all diagnostic factors of MetS.

Generally speaking, the compositions of gallstones are different between Western countries and Asia. In Western countries, cholesterol gallstones predominate, whereas pigment and mixed stone are more common in Asia [8]. Many studies in Western countries indicate that MetS is a risk factor for GSD [2,9]. However, in Taiwan, few studies have focused on this. A study which was performed during 2004 to 2005 also failed to show a significant association between MetS and GSD [8].

With the increasing incidence of MetS and a change in the Taiwanese dietary pattern, the relationships between metabolic factors and GSD must be explored. Therefore, the aims of this study were to establish whether there is an association between MetS and GSD in Taiwanese and analyze the relationships between metabolic factors and GSD.

## Methods

### Subjects and data collection

We conducted a cross-sectional study in the health examination center of Changhua Christian Hospital, Changhua City, Taiwan. Subjects who underwent a health examination during 2011 to 2012 formed our study population. The study protocol was approved by the Ethics Committee of Changhua Christian Hospital, Taiwan (CCH IRB No: 121113).

A total of 14,318 subjects who underwent physical examination, laboratory tests and abdominal sonography were enrolled. However, there were 2,073 subjects who had undergone a health exam in two successive years. The exclusion criteria also included a history of cholecystectomy (n = 163) and age <20 (n = 32). Finally, 12,050 subjects were included in our study.

Subjects were asked to complete a questionnaire that requested information on demographic data, age, gender, alcohol consumption, family history and past history (including diabetes mellitus, hypertension, chronic liver disease, etc.). Physical examination was also performed for our subjects and included body height, body weight, waist circumference, and blood pressure. Waist circumference (to the nearest 0.1 cm) was measured at the midpoint between the lower border of the rib and iliac crest. The blood pressure readings were obtained after subjects had sat down and rested for 10 min.

We collected blood data from participants after they had fasted overnight (> 8 hours). Data enrolled for analysis included age, fasting plasma glucose (FPG), triglyceride (TG), total cholesterol, high-density lipoprotein cholesterol (HDL-C), and low-density lipoprotein cholesterol (LDL-C).

### Diagnostic criteria

MetS was defined as the presence of three or more of the following five criteria proposed by the Taiwan National Health Department [10,11]: (1) abdominal obesity, defined as a waist circumference in men ≥ 90 cm and in women ≥ 80 cm; (2) hypertriglyceridemia: (higher TG) ≥ 150 mg/dL; (3) lower HDL-C: serum HDL-C < 40 mg/dL in men and < 50 mg/dL in women; (4) elevated blood pressure (systolic blood pressure ≥ 130 mmHg or diastolic blood pressure ≥ 85 mmHg or hypertension history (including self-reported or medical record); and (5) hyperglycemia: fasting plasma glucose (FPG) ≥ 100 mg/dL or DM history (including self-reported or medical record).

The diagnosis of GSD was determined based on the sonographic findings by an experienced gastrointestinal physician, who was unaware of the objectives of the study and blinded to the laboratory values. Gallstones were defined by the presence of strong intraluminal echoes that were gravity dependent or that attenuated ultrasound transmission (acoustic shadowing) in the gallbladder. We did not perform further analysis of gallstone composition.

Our sample population was formed from asymptomatic subjects who visited our health examination center. If the subjects had symptoms such as right upper abdominal pain and nausea, they were referred to the outpatient department and not be included in our study.

### Statistical analyses

To identify the risk factors for GSD, metabolic factors were further separated into categorical variables based on the definition of MetS in our study. All statistical analyses were conducted using SAS version 9.2. Student’s *t* test was applied to compare the differences between GSD and non-GSD groups for all continuous variables. The *X*^2^ test was used to compare the differences between GSD and non-GSD groups for all categorical variables. An age- and sex-adjusted logistic regression model was used to test the association between MetS and GSD. We also constructed univariate and multivariate logistic regression models to examine the associations between metabolic factors and GSD.

## Results

Among the 12,050 subjects, the prevalence of MetS was 27.20% (28.56% in men and 18.80% in women). The prevalence of GSD was 6.16% (6.15% in men and 6.17% in women). There were significant differences in the means of age, BMI, waist circumference, systolic blood pressure, diastolic blood pressure, HDL-C, TG and fasting blood glucose between the GSD and non-GSD groups (Table 1).

**Table 1:** Mean differences in continuous variables between cases and controls (mean ± SD)

**Variable**	**GSD (*n*** **= 734)**		**Non-GSD (*n *** **=11180)**		***P***
	**Mean**	**SD**	**Mean**	**SD**	
Age	52.7398	11.7932	46.9330	11.9712	< 0.0001
BMI	25.2892	3.6809	24.0816	3.6198	< 0.0001
Total cholesterol (TC)	191.8	37.2738	192.8	35.6617	0.4866
LDL-C	119.9	33.1849	119.9	31.0059	0.9784
Waist circumference	84.4238	10.0897	81.1618	9.9753	< 0.0001
Systolic BP	127.8	17.7644	122.6	17.1131	< 0.0001
Diastolic BP	81.0479	11.0727	78.8193	10.5878	< 0.0001
HDL-C	46.1391	12.1260	49.1702	12.7905	< 0.0001
Triglyceride (TG)	126.8	111.9	115.1	105.0	0.0039
FPG	100.3	24.2483	96.5119	21.3288	< 0.0001

Table 2 shows the crude (non-controlled) risks from the univariate analysis. Abdominal obesity, elevated blood pressure, hyperglycemia, lower HDL-C, and higher TG were all associated with a higher probability of GSD. However, there were no statistical differences between genders. Among the above-mentioned factors, abdominal obesity was most significantly and independently associated with GSD (OR = 1.88; Table 2).

**Table 2:** Comparison of metabolic factors between the GSD and non-GSD group

**Variable**	**GSD *n*** **= 741**		**Non-GSD *n*** **= 11182**		**OR**	**95% CI**	***P***
	***n***	**%**	***n***	**%**			
Sex							
Male	410	3.44	6253	52.48	0.9971	(0.8579-1.1589)	0.9697
Female	324	2.72	4927	41.35			
Abdominal obesity							
Larger	318	2.69	3260	27.59	1.8822	(1.6162-2.1920)	<0.0001
Normal	406	3.44	7834	66.29			
Systolic BP							
Higher (≥130 mmHg)	321	2.70	3463	29.11	1.7413	(1.4965-2.0261)	<0.0001
Lower (<130 mmHg)	410	3.45	7702	64.74			
Diastolic BP							
Higher (≥85 mmHg)	248	2.08	3062	25.74	1.3588	(1.1594-1.5924)	0.0001
Lower (<85 mmHg)	483	4.06	8103	68.12			
Elevated blood pressure							
Higher	412	3.46	4708	39.57	1.7603	(1.5141-2.0466)	<0.0001
Normal	321	2.70	6457	54.27			
HDL-C							
Lower	316	2.94	3510	32.66	1.5757	(1.3491-1.8404)	<0.0001
Normal	374	3.48	6546	60.92			
TG							
Higher	184	1.57	2367	20.15	1.2506	(1.0514-1.4874)	0.0114
Normal	538	4.58	8655	73.70			
Hyperglycemia							
Higher	250	2.15	2754	23.64	1.5975	(1.3617, 1.8740)	<0.0001
Normal	465	3.99	8183	70.23			

The odds ratio of MetS for GSD was 1.75 (95% CI = 1.42-2.15) in men and 2.46 (95% CI = 1.92-3.14) in women, and the overall odds ratio of MetS for GSD was 1.99 (95% CI = 1.70-2.33) (Table 3). When MetS was considered an independent factor, its association with GSD was still significant after adjusting for age and gender (OR = 1.61; 95% CI = 1.366-1.898; *P* < 0.0001).

**Table 3:** Association of metabolic syndrome (MetS) with gallstone disease by sex

**MS status**	**GSD**	**Non-GSD**	**OR (95% CI)**	***P***
	***n *** **(%)**	***n *** **(%)**		
**Male**				
Non-MetS	237 (5.14)	4378 (94.86)	1.75 (1.42-2.15)	< 0.0001
MetS	158 (8.64)	1670 (91.36)		
**Female**				
Non-MetS	203 (4.91)	3931 (95.09)	2.46 (1.92-3.14)	< 0.0001
MetS	107 (11.26)	843 (88.74)		
**Total**				
Non-MetS	440 (5.03)	8309 (94.97)	1.99 (1.70-2.33)	< 0.0001
MetS	265 (9.54)	2513 (90.46)		

Further considering what factors account most for GSD, multivariate logistic regression analysis showed that age (OR = 1.037; 95% CI = 1.029-1.046), waist circumference, (OR = 1.013; 95% CI = 1.004-1.023) and HDL-C (OR = 0.985; 95% CI = 0.977-0.993) were associated with GSD after adjusting for the other factors (Table 4).

**Table 4:** Age and metabolic risk factors for GSD by multiple logistic regression

	**Males**			**Females**			**All**		
	**OR**	**95% CI**	***P***	**OR**	**95% CI**	***P***	**OR**	**95% CI**	***P***
Age	1.040	(1.030-1.051)	< 0.0001	1.031	(1.018-1.044)	< 0.0001	1.037	(1.029-1.046)	< 0.0001
Waist circumference	1.014	(1.001-1.027)	0.0378	1.027	(1.012-1.043)	0.0006	1.013	(1.004-1.023)	0.0066
Systolic BP	1.004	(0.994-1.014)	0.4790	0.995	(0.985-1.006)	0.4005	0.999	(0.992-1.007)	0.8253
Diastolic BP	1.007	(0.992-1.022)	0.3450	1.013	(0.996, 1.030)	0.1486	1.008	(0.997, 1.020)	0.1506
HDL-C	0.969	(0.957-0.982)	<0.0001	0.988	(0.978, 0.999)	0.0330	0.985	(0.977-0.993)	0.0001
TG	1.000	(0.998-1.001)	0.4438	0.999	(0.997-1.001)	0.4585	0.999	(0.998-1.1001)	0.2685
FPG	0.999	(0.994-1.003)	0.5308	1.002	(0.997, 1.008)	0.4361	1.000	(0.997-1.004)	0.8728

Continuous variables: Age, Waist circumference, Systolic BP, Diastolic BP, HDL-C, TG, FPG.

To answer whether there were any differences in metabolic factors between genders, the multivariate logistic regression analysis showed that age, waist circumference, HDL-C were significantly and independently associated with GSD in both genders. Females had a higher odds ratio than males in waist circumference for GSD, whereas males had a lower odds ratio than females in HDL-C for GSD (Table 4).

## Discussion

The prevalence of GSD in our study was 6.16%. It accords with past studies, which reported the prevalence to be 3-11% in Taiwan [4,5,8]. The prevalence of GSD in Taiwan is a little lower than that in western countries (10-15%). The prevalence of MetS in our study subjects was about 27.20%, which is also in accordance with the current statistics from Taiwan [10,12].

Our study is the first Taiwanese study to show that the presence of MetS is associated with an increased risk of GSD (OR = 1.61; 95% CI = 1.366-1.898; *P* < 0.001) after adjusting for age and sex. Of all the factors of MetS, waist circumference and HDL-C were associated with GSD after adjusting for the other factors.

The metabolic syndrome seems to have 3 potential etiological categories: obesity and disorders of adipose tissue; insulin resistance; and a constellation of independent factors that mediate specific components of the metabolic syndrome. Other factors - aging, proinflammatory state, and hormonal changes - have been implicated as contributors as well [1]. The formation of GSD is related to bile acid metabolism, gallbladder motility, inflammation, and decreased bowel movement [7]. Factors that are related to GSD may affect any one of the above mechanisms. Factors of MetS, with the exception of hypertension, have also been reported as independent risk factors for GSD in many studies [4,5,7].

Our study suggests that gallstone formation increases with age, but not with gender. The more elderly had long-term exposure to many chronic factors, such as hyperlipidemia, alcohol consumption, and diabetes mellitus (DM). They may exhibit decreased motility of the gallbladder, thus causing GSD [4,5,13].

As in the other Asian studies [4,5,8], our study also indicates that gender is not related to GSD. Most previous western studies have shown that females are more likely to develop GSD than males [14,15]. It is generally believed that sex hormones are related to cholesterol metabolism, indicating that gender may be related to cholesterol stones [13]. However, pigment stones are often related to hemolysis, infection, and liver disease. The relationship between gender and GSD is probably different between Asia and Western population.

When adjusting for age and other metabolic factors, the present study revealed that waist circumference was associated with GSD. We also suggest that it is the most important metabolic factor for females with GSD. Obesity is a chronic inflammatory condition and is strongly linked to raised levels of pro-inflammatory factors [16]. It will increase liver secretion of cholesterol and make the bile supersaturated by increasing biliary secretion of cholesterol and causing GSD formation [8,13,17].

In our study, people with a higher blood pressure were more likely to have GSD than those with a normal blood pressure. However, after adjusting for age, sex and other metabolic factors, this likelihood was not significant. The mechanism by which abnormal blood pressure affects GSD is unclear. A previous study indicated that cholelithiasis in Asian obese patients is significantly associated with increased diastolic blood pressure. It is possible that people with hypertension may have more sympathetic nerve activities that easily inhibit bowel movement and cause GSD [18].

In line with previous research reporting lower HDL-C to be a risk factor for GSD [7,9], our study also arrived at this conclusion. Biliary cholesterol is mainly derived from HDL-C, with previous observations indicating that insulin resistance is associated with a low serum HDL-C concentration [19]. This may cause a high catabolic rate of HDL-C particles, which increases the rate of biliary cholesterol secretion [7,13,19].

In the present study, TG was related to GSD in the univariate analysis but not in the multiple logistic regression. One study indicated that in patients with higher TG, supersaturated bile may be related to the presence of obesity rather than to higher TG itself. However, the study also revealed that a higher TG level leads to decreased gallbladder contraction, which can easily lead to GSD [20]. The relationship between TG and GSD is complicated.

Whether DM is a risk factor for GSD remains controversial. While several western studies have suggested that DM is a risk factor for GSD, we here have showed that although hyperglycemia was more common in the GSD group in univariate analysis, this was not the case in the multiple logistic regression. Although study has shown that rats with hyperinsulinemia have the specific FOXO1 protein, which can increase the formation of cholesterol into bile [21]. Furthermore, hyperglycemia inhibits bile secretion from the liver and disturbs gallbladder contraction, and may affect gallbladder motility [13].

The current study indicates that lower HDL-C may be the most important metabolic factor for GSD in men, whereas in women, abdominal obesity had a higher odds ratio for GSD. It was previously found that women with abdominal obesity are prone to develop GSD [8]. The reason why HDL-C is the most important metabolic factor for GSD in men still requires further investigation.

Almost 10% of individuals with asymptomatic cholelithiasis in the general population can be expected to develop symptoms or complications that require treatment within five years. One study demonstrated that metabolic syndrome, diabetes and gallstone size were associated with complicated gallstone diseases [22,23]. For the above findings, we should pay attention to the relation between MetS and GSD.

There are several limitations to this study. First, the study design was retrospective and cross-sectional. Further longitudinal study is needed to clarify the true relationship between MetS and GSD. Second, subjects who underwent a health check-up were enrolled in the study. This may result in selection bias, such as the Hawthorne Effect. Furthermore, certain recognized GSD risk factors, such as a family history of GSD and weight loss history, were not included in the present study. Further research is needed to determine the true relationship between MetS and GSD considering the above factors. Research into Taiwanese gallstone composition can also be performed.

## Conclusions

This study found that MetS is related to GSD in the population of Taiwan. Waist circumference and HDL-C are associated with GSD. Men and women may possibly have different priorities and strategies to reduce the burden of GSD.

## Competing interests

No benefits in any form have been received or will be received from a commercial party related directly or indirectly to the subject of this article. The authors declare that they have no competing interests.

## Authors’ contributions

ICL proposed the study. CHC and ICL performed research, wrote the manuscript and contributed to discussion. YWY and JCL contributed to the conceptualizing the paper and Literature review. YLL contributed to the study design and data Interpretation. MFW and YHY collected and analyzed the data. All the authors read and approved the final manuscript.

## Pre-publication history

The pre-publication history for this paper can be accessed here:

http://www.biomedcentral.com/1471-2296/15/138/prepub

## References

[B1] Grundy SM Brewer HB Jr Cleeman JI Smith SC Jr Lenfant C Definition of metabolic syndrome: report of the National Heart, Lung, and Blood Institute/American Heart Association conference on scientific issues related to definition Circulation 2004 109 433 438 14744958 10.1161/01.CIR.0000111245.75752.C6

[B2] Grundy SM Cholesterol gallstones: a fellow traveler with metabolic syndrome? Am J Clin Nutr 2004 80 1 2 15213019 10.1093/ajcn/80.1.1

[B3] Mentes BB Akin M Irkörücü O Tatlicioğlu E Ferahköşe Z Yildinm A Maral I Gastrointestinal quality of life in patients with symptomatic or asymptomatic cholelithiasis before and after laparoscopic cholecystectomy Surg Endosc 2001 15 1267 1272 11727131 10.1007/s00464-001-9015-8

[B4] Liu CM Tung TH Chou P Chen VT Hsu CT Chien WS Lin YT Lu HF Shih HC Liu JH Clinical correlation of gallstone disease in a Chinese population in Taiwan: experience at Cheng Hsin General Hospital World J Gastroenterol 2006 12 1281 1286 16534886 10.3748/wjg.v12.i8.1281 PMC4124444

[B5] Chen CH Huang MH Yang JC Nien CK Etheredge GD Yang CC Yeh YH Wu HS Chou DA Yueh SK Prevalence and risk factors of gallstone disease in an adult population of Taiwan: an epidemiological survey J Gastroenterol Hepatol 2006 21 1737 1743 16984599 10.1111/j.1440-1746.2006.04381.x

[B6] Kratzer W Kachele V Mason RA Muche R Hay B Wiesneth M Hill V Beckh K Adler G Gallstone prevalence in relation to smoking, alcohol, coffee consumption, and nutrition. The Ulm Gallstone Study Scand J Gastroenterol 1997 32 953 958 9299677 10.3109/00365529709011208

[B7] Amigo L Zanlungo S Mendoza H Miquel JF Nervi F Risk factors and pathogenesis of cholesterol gallstones: state of the art Eur Rev Med Pharmacol Sci 1999 3 241 246 11261734

[B8] Lin YM Chen YH Hu NC Liao CS Chen JH Yang KC Shih CH The association of age, gender and metabolic factors with gallstone disease The Gastroenterological Journal of Taiwan 2011 28 11 18

[B9] Méndez-Sánchez N Chavez-Tapia NC Motola-Kuba D Sanchez-Lara K Ponciano-Rodríguez G Baptista H Ramos MH Uribe M Metabolic syndrome as a risk factor for gallstone disease World J Gastroenterol 2005 11 1653 1657 15786544 10.3748/wjg.v11.i11.1653 PMC4305948

[B10] Tsai TY Cheng JF Lai YM Prevalence of metabolic syndrome and related factors in Taiwanese high-tech industry workers Clinics 2011 66 1531 1535 22179154 10.1590/S1807-59322011000900004 PMC3164399

[B11] Ministry of Health and Welfare The definition of the metabolic syndrome in adults Available online: http://www.hpa.gov.tw/BHPNet/Web/HealthTopic/TopicArticle.aspx?id=200712250123&Class=2&parentid=200712250023. Accessed 2014 JUL.30

[B12] Hwang LC Bai CH Chen CJ Prevalence of obesity and metabolic syndrome in Taiwan J Formos Med Assoc 2006 105 626 635 16935763 10.1016/S0929-6646(09)60161-3

[B13] Chen LY Qiao QH Zhang SC Chen YH Chao GQ Fang LZ Metabolic syndrome and gallstone disease World J Gastroenterol 2012 18 4215 4220 22919256 10.3748/wjg.v18.i31.4215 PMC3422804

[B14] Lirussi F Nassuato G Passera D Toso S Zalunardo B Monica F Virgilio C Frasson F Okolicsanyi L Gallstone disease in an elderly population: the Silea study Eur J Gastroenterol Hepatol 1999 11 485 491 10755250 10.1097/00042737-199905000-00004

[B15] Tazuma S Gallstone disease: epidemiology, pathogenesis, and classification of biliary stones (common bile duct and intrahepatic) Best Pract Res Clin Gastroenterol 2006 20 1075 1083 17127189 10.1016/j.bpg.2006.05.009

[B16] Dixon JB O’Brien PE Obesity and the white blood cell count: changes with sustained weight loss Obes Surg 2006 16 251 257 16545154 10.1381/096089206776116453

[B17] Heaton KW Braddon FE Emmett PM Mountford RA Hughes AP Bolton CH Why do men get gallstones? Roles of abdominal fat and hyperinsulinemia Eur J Gastroenterol Hepatol 1991 3 745 751

[B18] Liew PL Wang W Lee YC Huang MT Lin YC Lee WJ Gallbladder disease among obese patients in Taiwan Obes Surg 2007 17 383 390 17546848 10.1007/s11695-007-9068-4

[B19] Karhapää P Malkki M Laakso M Isolated low HDL cholesterol. An insulin-resistant state Diabetes 1994 43 411 417 8314013 10.2337/diab.43.3.411

[B20] Smelt AH Triglycerides and gallstone formation Clin Chim Acta 2010 411 1625 1631 20699090 10.1016/j.cca.2010.08.003

[B21] Kovacs P Kurtz U Wittenburg H Hepatic insulin resistance ties cholesterol gallstone formation and the metabolic syndrome Ann Hepatol 2008 7 262 264 18753998

[B22] Halldestam I Enell EL Kullman E Borch K Development of symptoms and complications in individuals with asymptomatic gallstones Br J Surg 2004 91 734 738 15164444 10.1002/bjs.4547

[B23] Ata N Kucukazman M Yavuz B Bulus H Dal K Ertugrul DT Yalcin AA Polat M Varol N Akin KO Karabag A Nazligul Y The metabolic syndrome is associated with complicated gallstone disease Can J Gastroenterol 2011 25 274 276 21647463 10.1155/2011/356761 PMC3115009

